# Impulse Magnetization of Nd-Fe-B Sintered Magnets for Sensors

**DOI:** 10.3390/s16040569

**Published:** 2016-04-21

**Authors:** Marek Przybylski, Dariusz Kapelski, Barbara Ślusarek, Sławomir Wiak

**Affiliations:** 1 Tele & Radio Research Institute, Ratuszowa 11 St, 03-450 Warsaw, Poland; dariusz.kapelski@itr.org.pl (D.K.); barbara.slusarek@itr.org.pl (B.Ś.); 2 Lodz University of Technology, Institute of Mechatronics and Information Systems, Stefanowskiego 18/22 St, 90-924 Lodz, Poland; swiak@wp.pl

**Keywords:** Nd-Fe-B permanent magnets, permanent magnets, impulse magnetization, magnetic saturation, magnetic induction, coercivity

## Abstract

Magnetization of large Nd-Fe-B sintered permanent magnets is still challenging. This type of permanent magnet is electrically conductive, so impulse magnetization causes a flow of eddy currents which prevent magnetization of the whole volume of the magnet. The paper deals with the impulse magnetization of sintered Nd-Fe-B permanent magnets and shows a method for the determination of suitable parameters for the supply system. The necessary magnetic field strength for magnetization of the magnet to saturation was determined. The optimal magnetizing fixture supply voltage for magnetization to saturation was determined from simulations in PSpice software, finite element analyses in Maxwell 15 and measurements. Measurements of magnetic induction on the surface of the Nd-Fe-B magnet are also presented to ensure that a magnet with 70 mm diameter and 20 mm in height is fully saturated.

## 1. Introduction

Sintered Nd-Fe-B permanent magnets make it possible to increase the performance of devices and can also enable their miniaturization. The main field of application for this type of permanent magnet are electric machines such as electric motors and generators. For example, in direct current motors permanent magnets are applied in stators as an excitation source of magnetic field. In brushless direct current motors permanent magnets are applied to rotors. In small wind power plants permanent magnets are used in 3-phase axial flux machines, without a soft magnetic core, and with power of about a few kW. With the development of magnetic materials the range of their application is broadening, among others in magnetic sensors. Developments in material engineering allow the elaboration of new materials with improved parameters and their application in a new generation of magnetic sensors. For many applications the diversity of the physical properties of the materials and the possibility of producing magnets with a complicated shape with two or more magnetic poles are very important and enable the application of magnets in magnetic sensors. These types of sensor are elements of many measuring devices. The main role of magnetic sensors is not only in the measurement of the distribution and parameters of the magnetic field, but also in the measurement of another physical quantities such as magnetic force, where the magnetic field transforms into a processing measurement signal. For many applications it is very important to magnetize to saturation the whole volume of the magnet and ensure a suitable distribution of magnetic induction on the surface of the permanent magnets in magnetic sensors [1,2].

Magnetic sensors with permanent magnets are often applied in measuring transducers, such as devices for the measurement of linear or rotational speed, in tachometers, in reeds, vibrations and pressure meters, *etc.* Three pairs of permanent magnets are used in a three degrees of freedom displacement measurement system to create magnetic field in the air. This magnetic field is measured by Hall sensors and their signals give position information. In this type of device, permanent magnets must be magnetized uniformly for accurate position measurement [3]. In turn, a permanent magnet is used in a noncontact force measuring system and must also be magnetized uniformly. In the case of non-uniform magnetization, the force will be less than expected in the design stage of the sensor because a permanent magnet produces less magnetic flux [4]. Permanent magnets are also used in electromagnetic flow meters for liquid metals. The converter consists of a contactless electromagnetic pump with a torque sensor mounted on the pump shaft. The electromagnetic pump is composed of two rotating steel discs with embedded permanent magnets with alternating poles. The rotation of the discs creates a travelling sinusoidal magnetic field and eddy currents within the liquid metal. The interaction of the magnetic field and induced eddy currents generates electromagnetic Lorentz force that provides the pumping effect. The flow rate is proportional to this force, and the torque is measured by a torque sensor and is converted to a flow rate. In this meter, uniform magnetization is essential for trustworthy flow measurement of the liquid metal, especially for pipes with large diameters and thicknesses [5].

Sintered Nd-Fe-B magnets have high magnetic energy and this kind of permanent magnet is mainly magnetized in the last stage of production using high voltage (about 5 kV) impulse magnetizers equipped with capacitors with large capacitance [6]. The electric energy stored in the capacitors is discharged by a magnetizing fixture, creating the magnetic field needed for magnetization.

Each hard magnetic material has a defined magnetic field strength that enables its magnetization to saturation. Producers of permanent magnets do not often provide such information. If they do provide the value of this magnetic field strength, often it is more than practically needed. A larger magnetic field causes higher electric current impulses and the magnetization fixture is mechanically strained more than necessary. This can mechanically and thermally spoil the magnetization fixture earlier than expected.

Magnetic, electric, electromagnetic and electromechanical devices need permanent magnets with different numbers and configurations of magnetic poles. Larger magnets with two or more magnetic poles can be composed of two pole magnets. However, in this situation there are many problems, e.g., gluing of magnets, or imperfect distribution of magnetic flux density in the air gap. A magnetizing fixture should be designed and made for the required dimensions of the magnet, number of magnetic poles and their patterns. Sometimes a magnetizing fixture can be universal for some types of permanent magnets, such as two pole with axial magnetization. Permanent magnets with different dimensions can be magnetized in this type of fixture. The amount of electrical energy gathered in a bank of capacitors determines the size and weight of the permanent magnets to be magnetized.

Nd-Fe-B sintered permanent magnets are made of electrically conductive material. That is why in these kinds of magnet, during impulse magnetization, eddy currents flow and cause inhomogeneous magnetization, power loss and unwanted heating of the magnets. This can be mainly seen in magnets with a large volume.

Impulse magnetizers are built as low voltage (450–500 volts), medium voltage (800–1000 volts) and high voltage (1500–3000 volts) appliances. Higher voltage magnetizers produce shorter magnetic field impulses and are more efficient. A process of impulse magnetization of magnets using condensers was first applied in about 1944, but there was a problem with such magnetizers. They often produced, depending on the resistance of the magnetizing fixture, oscillating currents which in a second pulse causes demagnetization of the magnet [6]. Improved magnetizers were applied from 1954 [7]. Two-pole magnets can be magnetized axially or diametrally in the case of cylindrical and ring magnets. Permanent magnets can be magnetized before or after mounting in a device [8,9]. In the case of ring magnets, they can be magnetized radially, also called homopolarly (one pole on an internal diameter and the other on an external diameter) [10]. Multipolar magnets can be magnetized radially or axially. The magnetization of a bush magnet with four radial poles was analyzed in [11,12,13,14]. In turn, the magnetization of an axial eight-pole magnet was analyzed in [15,16], and six-pole in [17].

First, for an analysis of the impulse magnetization process, analytical calculations were used. Since the 1980s, with improved computers, numerical analysis has been introduced in the design of magnetizers and the features of impulse magnetization. Nakata and Takahashi performed the first analyses of a transient magnetic field in a capacitor-discharge impulse magnetizer. They combined Maxwell’s equation with Kirchhoff’s equation for an electromagnetic analysis of the magnetization of a four-pole polymer-bonded magnet [11]. However, they did not consider eddy currents in the magnet, but took into account the eddy currents in the pole piece and yoke of a magnetizing fixture. Lee in his article deals with the magnetization of an axially oriented eight-pole epoxy-bonded Nd-Fe-B magnet. This type of magnet has high resistivity and in these magnets eddy currents can be neglected. However, it was also assumed that the magnet has a permeability equal to 1 and this was represented as an air gap in simulations [15]. This assumption is valid only for highly saturated permanent magnets. Jewell, Howe and Birch analyzed the magnetization of a four-pole radial Nd-Fe-B permanent magnet for motors [12]. In this research, they took eddy currents into consideration in magnetizing a bush magnet, but the magnet was only 1.5 mm thick and the eddy currents could also be neglected. A simulation of magnet magnetization was also conducted in-situ in the vicinity of soft magnetic materials, so the current required was not as high as in an air-core magnetizing fixture. Magnet magnetization in a mounted motor is difficult also because of the existence of bulk soft magnetic materials in a motor. The same authors conducted magnetization and measurements of electric parameters. Magnetization of a fully mounted motor is difficult and full saturation is hard to obtain because of the insufficient capacitance of the capacitors [13,14]. Heiden, Arkadan and Brauer also reported in [18] that magnetization of an Nd-Fe-B magnet, with the use of a magnetization fixture with a soft magnetic core, was not fully conducted due to eddy currents induced in the magnet. Air-cored magnetizing fixtures are easier to analyze because the inductance of the magnetizing winding is linear and an impulse current can be closely calculated. A four-pole radial field magnetizing fixture was applied for magnetization of an isotropic Nd-Fe-B bonded magnet. Because of the high resistivity of the magnet, impulse eddy currents in this magnet were neglected [19]. An axial field multipole magnetizing fixture was designed and used for magnetization of a six-pole magnet. This type of magnetizing fixture is air-cored and 24 kA peak currents are produced [17]. Magnetization of an eight-pole planar Nd-Fe-B ring bonded magnet was analyzed and a magnetizing fixture for this magnet was designed and applied. This fixture uses soft magnetic composite (SMC) material for the magnetic core. Due to the high resistivity of the SMC material, it was possible to decrease the impulse magnetizing currents in comparison with air-cored fixtures. The SMC material enabled uniform magnetization of the core and the lack of displacement of magnetic flux from the magnetic core [16,20]. Multipolar magnetization of a planar and cylindrical permanent magnet with 36 magnetic poles resembling a chess-board was developed. These magnets could be applied in rotary linear actuators and planar electric motors [21,22]. Recently, for a description of the magnetization process of permanent magnets, the finite element method has been used with the Jiles-Atherton hysteresis model [23].

In the paper the magnetization process of an Nd-Fe-B permanent magnet is considered and optimized electrical supply parameters were determined for magnetization of the magnet practically to saturation. Impulse currents with low values enable electrical energy savings during magnetization of permanent magnets, especially in mass production. What is more, it is possible to increase the number of magnets magnetized in the same amount of time, because energizing the capacitors to a lower voltage takes less time.

## 2. Materials and Methods

The research was conducted on commercially available grade N38 anisotropic sintered Nd-Fe-B permanent magnets. These permanent magnets, according to supplier data, have the following parameters: remanence B_r_ = 1.25 T, coercivity of magnetic flux density min. H_Cb_ = 899 kA/m, coercivity of magnetic polarization min. H_cJ_ = 955 kA/m, maximum value of (BH) product (BH)_max_ = 310 kJ/m^3^, resistivity ρ = 1.44 × 10^−6^ Ω·m [24]. A permanent magnet 70 mm in diameter and 20 mm in height was chosen for the analysis of impulse magnetization.

For determination of the saturation magnetic field, samples made of N38 material with dimensions 30 × 10 × 10 mm with the direction of anisotropy according to 10 mm length were used. It was not possible to measure the parameters of the magnet 70 mm in diameter and 20 mm in height that was chosen for this study, due to the limitations of the measuring equipment. Measurements of magnetization and demagnetization curves were conducted using a hysteresisgraph AMH-20K-HS manufactured by Laboratorio Elettrofisico Walker LDJ Scientific (Nerviano, Italy). Measurements were conducted based on IEC standard IEC 60404-5.

Impulse magnetization was conducted using an impulse magnetizer. A magnetization fixture was supplied from an impulse magnetizer, designed and manufactured in the Tele & Radio Research Institute, that consists of a bank of capacitors with C = 1 mF capacitance and regulated voltage U_c_ from 0 to 5000 V. The impulse current is triggered by a thyristor as an electronic switch. The impulse magnetizer is also equipped with a diode that enables an aperiodical shape of current waveforms *vs.* time in the winding. The impulse magnetizer is supplied from an AC 230 V, 50 Hz line.

The magnetizing fixture was designed and manufactured by Wroclaw University of Technology. It is a cylinder with an internal diameter of 80 mm for a magnet to be magnetized and an external diameter of 150 mm and 193 mm in height. The magnetization fixture consists of an internal copper coil with 440 turns of 3 mm diameter wire and an external tube of aluminium with a diameter of 150 mm and 4 mm thickness to ensure high strength against high impulse currents and forces. The aluminium tube helps to increase the mechanical strength of the coil and allows easier emission of the heat that comes from the currents in the wires during frequent magnetization. This tube is paramagnetic and is sufficient for the mechanical safety of the coil. The coil and the tube have the same axis of rotation.

## 3. Results

### 3.1. Determination of Saturation Magnetic Field

**Figure 1:**
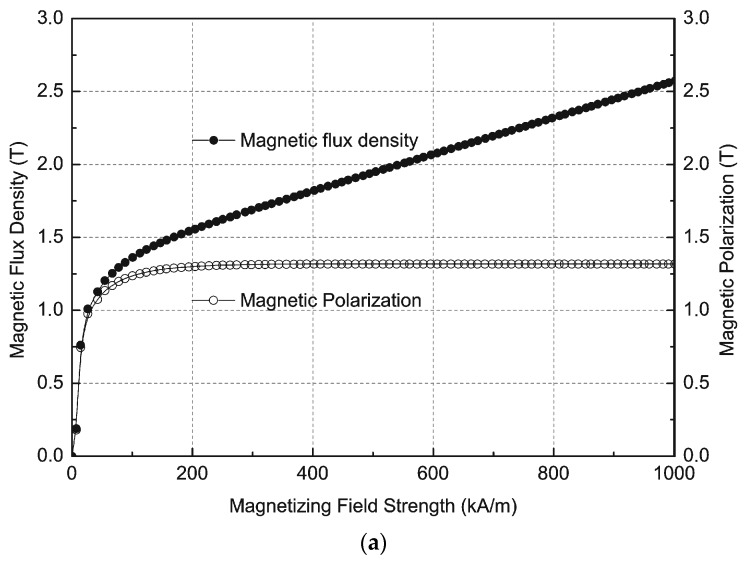

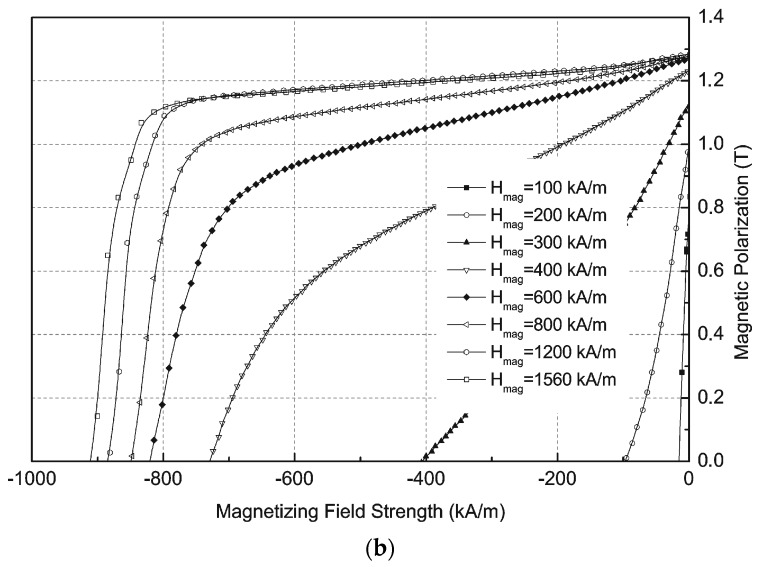
Measurements of (**a**) magnetic flux density as a function of magnetic field strength B = f(H) and magnetic polarization as a function of magnetic field strength J = f(H) magnetization curves; (**b**) J = f(H) demagnetization curves of an Nd-Fe-B permanent magnet.

**Figure 2:**
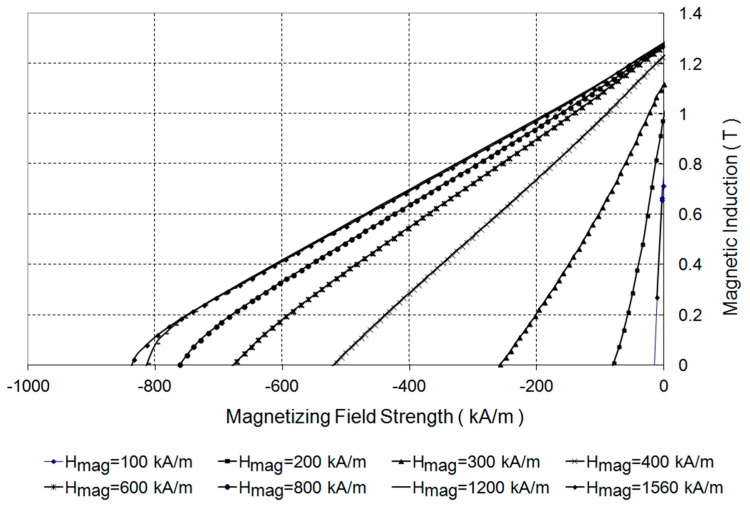
Measurements of B = f(H) demagnetization curves of an Nd-Fe-B permanent magnet.

Figure 1a shows the magnetization curves B = f(H), magnetic flux density as a function of magnetic field strength and J = f(H), and magnetic polarization as a function of the magnetic field strength for the maximum magnetizing field strength H_max_ = 1000 kA/m. Measurements of the demagnetization curves are presented in Figure 1b and Figure 2.

From the demagnetization curves it is possible to determine the parameters of permanent magnets such as remanence B**_r_**, coercivities H_cB_, H_cJ_ and maximum energy product (BH)_max_. Figure 1b presents the demagnetization curves J = f(H) for magnetic field strengths from the H_mag_ = 100 to 1560 kA/m range. The magnetization direction was parallel to the anisotropy direction. The permanent magnets were thermally demagnetized after each measurement cycle. Figure 2 shows the results of measurements of the second quadrant of the hysteresis loop of an Nd-Fe-B permanent magnet with different magnetizing field strengths.

Figure 2 shows that the second quadrant’s characteristic magnetic induction values as a function of the magnetic field strength of the magnet when magnetized with 1200 kA/m and more overlap, and this ensures the distribution of magnetic induction on a surface with the same values. This means that a magnetization field strength of 1200 kA/m is enough for magnetization to saturation.

**Figure 3:**
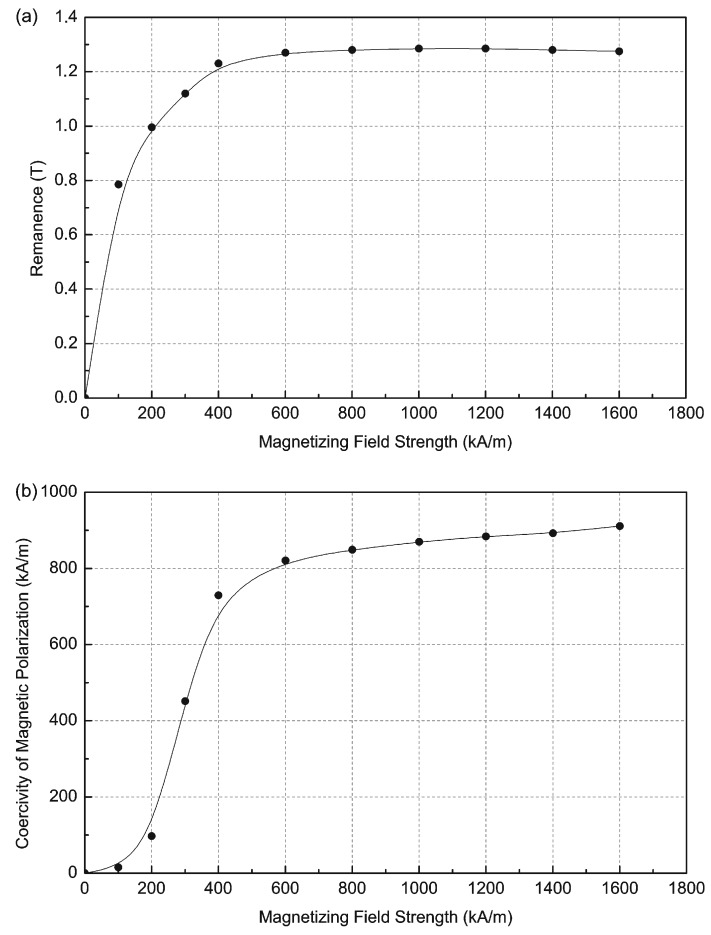
Measurements of (**a**) magnetic remanence Br and (**b**) coercivity of magnetic polarization H_cJ_ as a function of magnetizing magnetic field strength H_mag_.

Figure 3 shows the measurements of remanence and coercivity of a sintered Nd-Fe-B permanent magnet as a function of the magnetization field strength. The results show that the remanence for magnetizing fields greater than 600 kA/m does not increase. The coercivity of the samples increases with increasing magnetizing field strength, but from the value of magnetizing field strength of 600 kA/m increases only slowly. The coercivity, as shown in Figure 3b with increasing magnetization field from 1000 kA/m to 1600 kA/m, increases only by about 4.7%, while the increase of the magnetization field is about 60%. When all the magnetization field vectors are parallel to the external magnetic field, then the magnet is magnetized to saturation. 

### 3.2. Impulse Magnetizer and Magnetization Fixture

**Figure 4:**
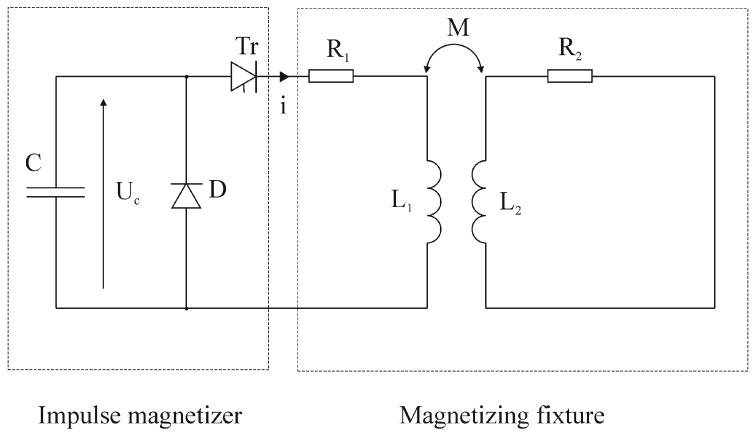
Electric diagram of the impulse magnetizer with a magnetizing fixture.

The analysis of the impulse magnetization process of permanent magnets made of sintered Nd-Fe-B alloys was conducted in several steps. These steps led to the determination of the supply parameters that enable magnetization of the magnet to saturation in the whole volume, not only on the surface. The first step was to calculate the magnetization fixture’s electrical parameters from the dimensions and materials of the fixture. Because of the existence of the aluminium tube, the magnetizing fixture must be considered as a coreless transformer with a short-circuited secondary winding and cannot be treated as an air coil. This presents some difficulties in the analysis of the current in the magnetizing fixture’s coil. What is more, eddy currents induced in the aluminium tube cause an opposite magnetic field which decreases the magnetic field inside the magnetizing fixture. Figure 4 presents an electric diagram of the magnetizer with a magnetizing fixture.

R_1_ and L_1_ represent the resistance and self-inductance of the coil. R_2_ and L_2_ represent the resistance and self-inductance of the aluminium tube. The magnetic coupling between the coil and aluminium tube is represented by mutual inductance M. The resistance of the coil was measured and R_1_ = 0.38 Ω. Values of self, mutual inductances and resistance of the aluminium tube were calculated from the dimensions and number of turns of the magnetizing fixture and are equal to L_1_ = 5.52 mH, R_2_ = 17.10 μΩ, L_2_ = 73.22 nH, M = 11.40 μH. These parameters were essential for subsequent analyses with PSpice software.

### 3.3. Analysis of Impulse Magnetization of Nd-Fe-B Magnet

**Figure 5:**
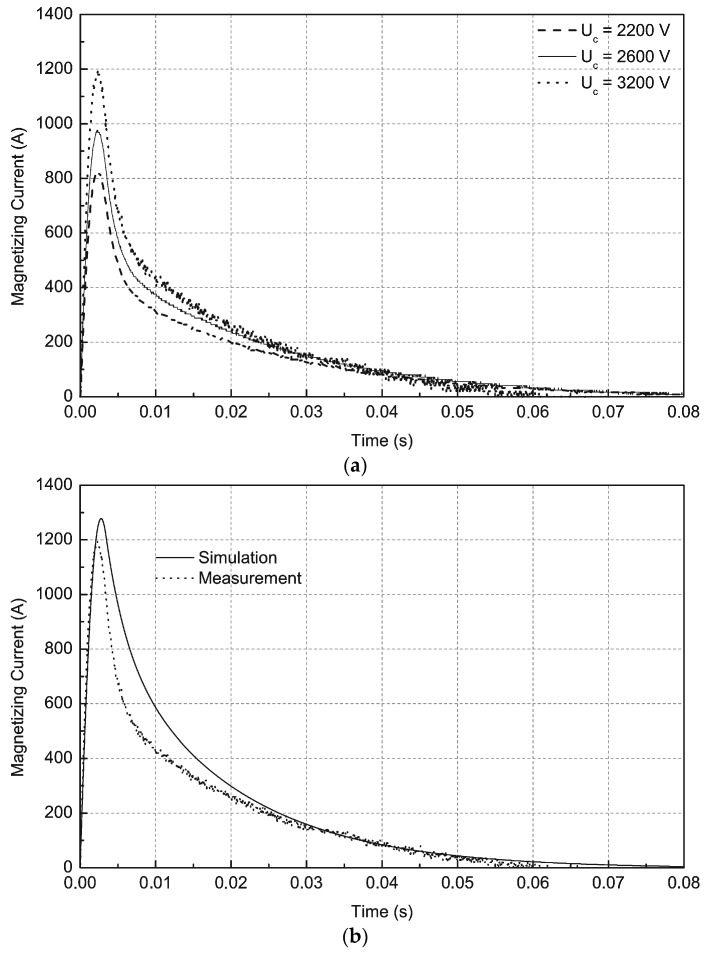
(**a**) Measurements of impulse magnetization currents for different voltages; (**b**) comparison of impulse magnetization currents for voltage U_c_ = 3200 V.

Next a series of numerical calculations were conducted in PSpice software, which is an electric circuit simulator that uses the electrical parameters of elements, such as resistance, inductance and capacitance. Measurements of currents in the coil for impulse magnetization were compared with PSpice calculations. As an example, Figure 5a shows the measurements of the impulse currents in the coil of the magnetization fixture. Measurements of current waveforms were conducted using a Tektronix TDS 210 oscilloscope (Beaverton, OR, USA) with a Tektronix A 621 AC current probe. Figure 5b shows the comparison of the measurement and simulation of the magnetizing current for U_c_ = 3200 V. The differences between the measurement curve and the simulation curve are caused by inaccuracy in determining parameters such as the resistance of the aluminium tube R_2_, inductances L_1_ and L_2_, and mutual inductance M. Further inaccuracy is a result of the changing parameters of the capacity of capacitor C with time, and the definition of thyristor Tr as an ideal element in PSpice software. These parameters were used in simulations in PSpice software.

**Figure 6:**
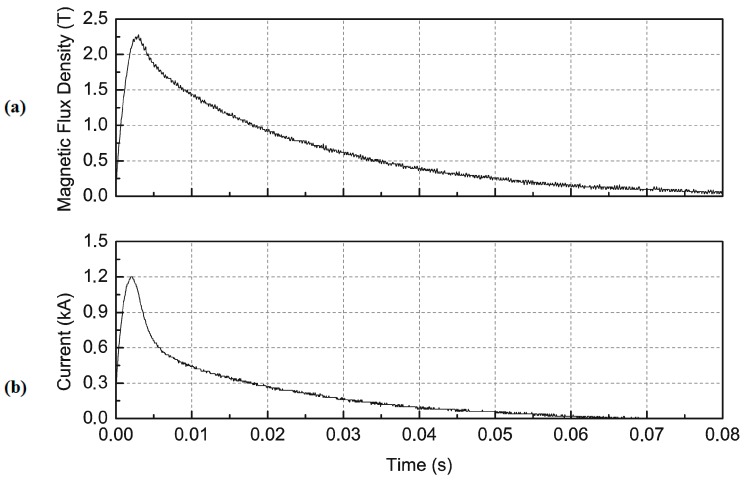
Measurements of (**a**) magnetic flux density in the middle of magnetization coil and (**b**) current, for U_c_ = 3200 V magnetization voltage as a function of time [25].

The impulse magnetizer with capacitors charged to 3200 V creates in the magnetizing fixture an impulse current with a maximum I_m_ = 1200 A (Figure 6b). This current produces an impulse of magnetic flux density in the middle of the magnetizing fixture equal to B_m_ = 2.28 T (Figure 6a), measured by a fluxmeter with a THS 119 Hall sensor (Toshiba, Tokyo, Japan), with a supply circuit [25]. The curves in Figure 6 show that the magnetic induction increases more slowly than the current and decreases for longer than the current. This is caused by eddy currents induced in the aluminium shell of the coil.

**Figure 7:**
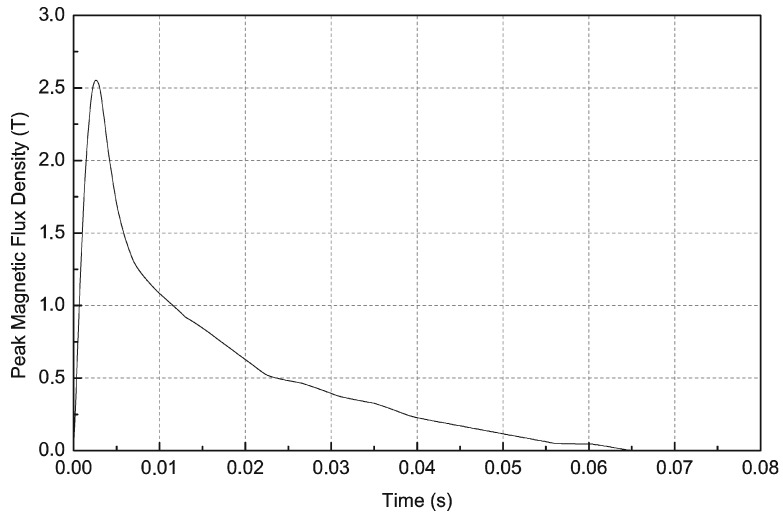
Simulation of magnetic flux density as a function of time in the middle of magnetizing coil in Maxwell 15.

Computer calculations were performed for comparison in the Maxwell 15 finite elements method (FEM) software. The course of the current from upper Figure 6 was implemented in FEM software as the source of magnetic field. The simulated time course of magnetic induction in the middle of the coil is presented in Figure 7. Calculations were performed in two dimensions because the magnetizing fixture has axial symmetry. The maximum value of magnetic induction is equal to 2.56 T. The course is not smooth because the current course implemented in FEM software is composed from only 20 points due to Maxwell 15 limits.

The difference between the measured and simulated maximum values of induction is about 12%. This may be caused by numerical errors in the FEM software and measurement errors in the THS 119 Hall sensor fluxmeter.

**Figure 8:**
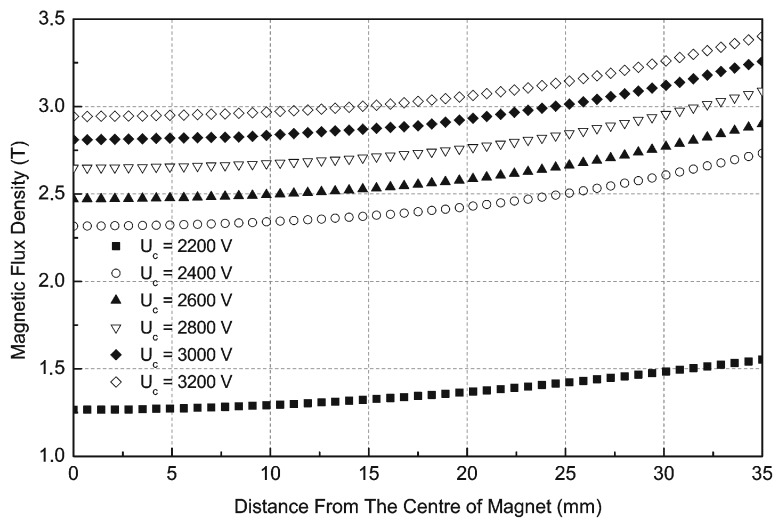
FEM simulations of magnetic induction in the Nd-Fe-B sintered magnet for different capacitor voltages, time t = 2.6 ms.

The measured current characteristics i(t) presented in Figure 5a, with different supply voltages, were exported to the FEM software as the supply currents. Numerical calculations of the magnetic flux density in the 70 mm diameter 20 mm high magnet were performed and are presented in Figure 8. The magnetic flux density in the centre of the magnet for U_c_ = 2800 V is equal to 2.60 T, which is practically sufficient for magnetization to saturation according to Figure 3a,b.

**Figure 9:**
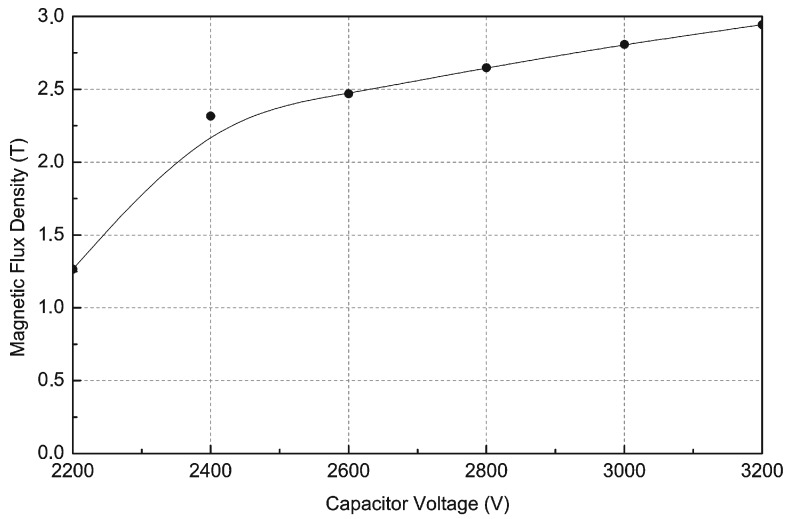
FEM **s**imulations of magnetic induction in the centre of an Nd-Fe-B magnet as a function of capacitor voltages.

Figure 9 shows the magnetic induction in the middle of the Nd-Fe-B permanent magnet for different supply voltages of the magnetizer. This curve is nonlinear to capacitor voltage 2600 V and above this value the magnetic induction increases linearly.

The computer simulations presented in Figure 9 show that for voltage U = 2800 V, the magnetic induction inside the magnet B = 2.65 T, which is practically sufficient for magnetization to saturation of an Nd-Fe-B type N38 permanent magnet.

**Figure 10:**
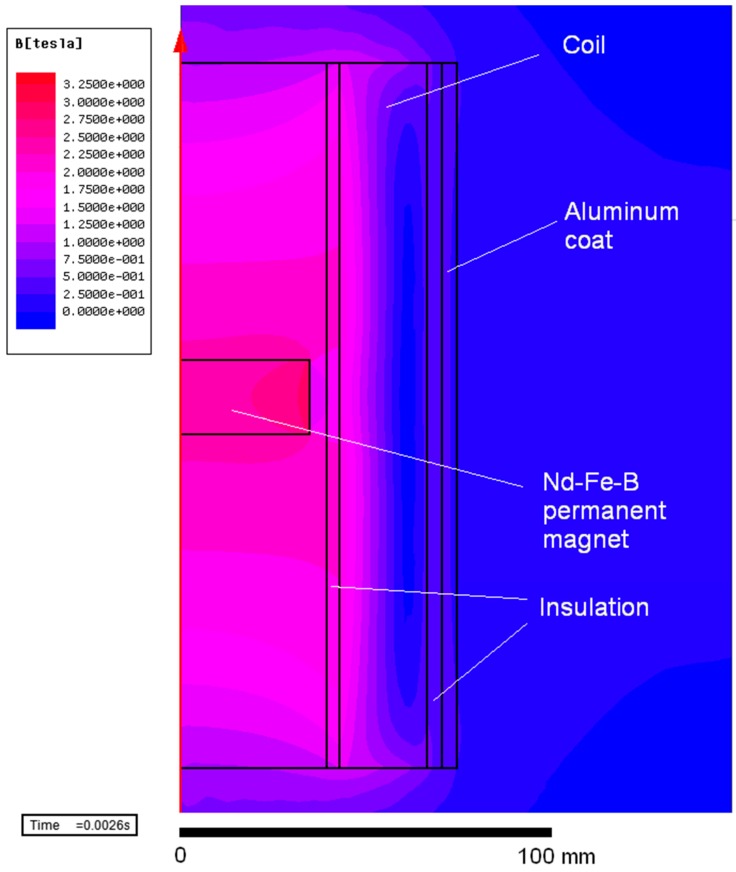
FEM simulations in magnetizing fixture and Nd-Fe-B magnet for U_c_ = 2800 V, I_m_ = 1032 A, t = 2.6 ms.

Figure 10 shows the magnetic flux density in the magnetizing fixture and in the magnet during impulse magnetization. For this voltage, the magnetic flux density on the edge of the magnet is equal to 3.10 T. We can observe the influence of eddy currents on the magnetic flux density distribution in the magnet.

### 3.4. Measurements of Magnetic Induction on the Surface of the Magnet

**Figure 11:**
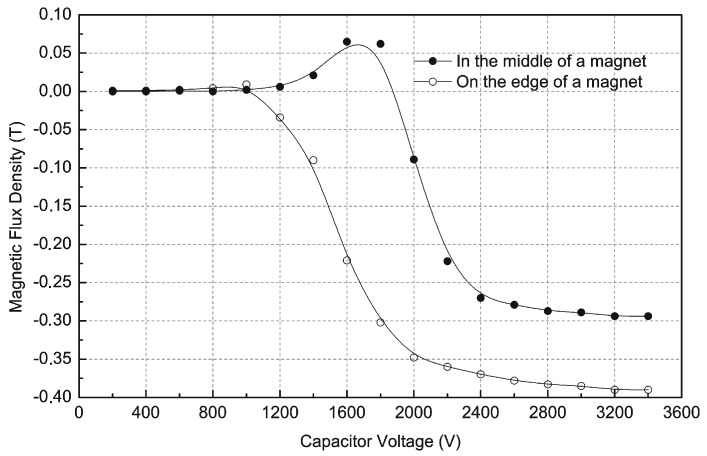
Measurements of magnetic induction on the surface of the magnet, for different capacitor voltages.

Measurements of induction on the surface of the cylindrical magnet (70 mm diameter, 20 mm height) in the middle of the magnet and 5 mm from the edge of the magnet made of Nd-Fe-B material are shown in Figure 11 for different capacitor voltages. Measurements were conducted using a model 5070 teslameter made by F.W. Bell (Orlando, FL, USA), equipped with an SAH57-1904 axial probe. These curves also show that the capacitor voltage U_c_ = 2800 V is practically sufficient for magnetization of the magnet to saturation.

**Figure 12:**
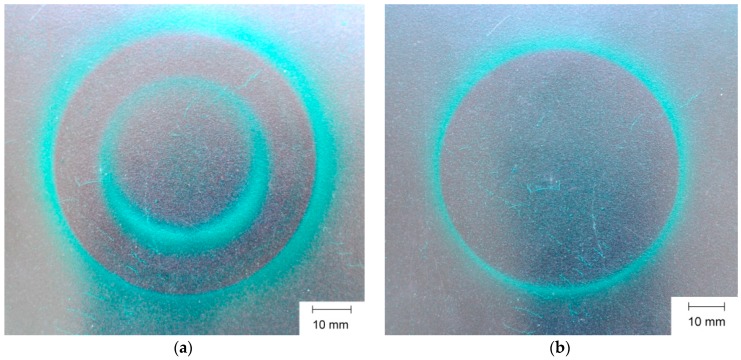
Photos of magnetic poles on the surface of N38 type Nd-Fe-B magnet for magnetization voltages (**a**) for 1600 V and (**b**) for 2800 V.

Photos were also taken of the magnetic field on the surface of the Nd-Fe-B permanent magnet. Figure 12 shows photos of the magnetic poles of the magnet displayed on a magnetic field viewing film—a special foil with nickel particles suspended in oil for observation of magnetic poles. In Figure 12a it is possible to see two poles of magnetic field, the inner bright circle dividing the magnet on two opposite poles. The values of the magnetic induction can be taken from Figure 6 for U_c_ = 1600 V.

## 4. Conclusions

Measurements of magnetic induction on the surface of the magnet show that for the applied voltage of U_c_ = 2800 V in the magnetizer to the capacitors, the magnet is practically saturated. Higher voltages do not increase the saturation of the magnet, but rather just cause higher energy costs, and a longer process of magnetization due to energizing of the capacitors and higher heating of the magnetization coil. Measurements confirming the numerical calculations and parameters of the electrical supply are determined optimally. The described method can be useful for producers of permanent magnets in the design and production of magnets with different shapes and dimensions. This method can also be useful for determining the magnetizing parameters of new types of permanent magnets, especially with low resistivity and high coercivity, when magnetized with very fast impulses of magnetic field.
